# The Fundamental Fallacy of “Empathic AI”

**DOI:** 10.1002/hast.5011

**Published:** 2025-06-25

**Authors:** Karola Kreitmair

**Keywords:** artificial intelligence, chatbots, recognition, empathy, patient‐clinician relationship, bioethics

## Abstract

*“Empathic AI” is being adopted in clinics as a means of offloading some of the work of clinician‐patient encounters. Indeed, a recent study reported that generative large language models such as GPT4 were perceived as being more empathetic than human physicians. I argue that encounters between AI chatbots and patients lack an essential feature of good clinical encounters*—recognition. *More fundamental than empathy, Hegelian recognition is a precondition for features such as honesty and respect for autonomy that are central tenets of medical ethics. I argue that patients have a* justified expectation *of mutual recognition in a clinical encounter and that, given specific limitations of AI chatbots, this justified expectation cannot be met by them. Problematically, however, AI chatbots are designed to mimic human expressions of recognition, resulting in an alienating absurdity at the heart of “empathic AI.” This fundamental incoherence is not merely a philosophical curiosity; it is an issue that must be directly addressed if AI chatbots are to take on roles in clinical encounters*.

## Article

In a recent study published in *JAMA Internal Medicine*, researchers compared human clinicians to an artificial intelligence (AI) chatbot—a generative large language model (LLM), that is—trained to respond to health care questions.^1^ The researchers presented the human clinicians and the AI chatbot with a set of 195 medical questions sourced from Reddit. Medical professionals then blindly rated the responses of the human clinicians and the AI chatbot. Not only did they rate the AI chatbot responses as qualitatively better; they also rated them as significantly more empathetic than those of their human counterparts. What can we conclude from this? Perhaps it is a nudge for clinicians to improve their communication. But can we also conclude that AI chatbots like ChatGPT or GPT4 are more empathetic than human clinicians? The answer, of course, is no. AI chatbots are not more empathetic than human clinicians because AI chatbots are not empathetic, period.

The previous statement may seem obvious, but in much of the discourse around the adoption of AI chatbots into health care interactions, this obvious fact is obscured and denied in ways that I argue are problematic. My focus in this article will not be empathy directly but a neighboring concept that is more elementary than empathy. This is the concept of *recognition*.^2^ I will return to the relationship between recognition and empathy later. First, I will explain what I mean by “recognition.” I will show that recognition is an essential feature of (most) good clinical encounters and that it is necessarily absent in an encounter between a patient and an AI chatbot, rendering (most) encounters between patients and AI chatbots inadequate. After describing three necessary conditions of recognition, I will explain why patients are justified in their expectation of engaging in recognition in a clinical encounter. I will consider two objections to my view before closing with some thoughts on appropriate uses of AI chatbots and on the ethics of the use of “empathic AI” with patients who lack competency or capacity.

## What Is Recognition?

First, let me say what I do not mean by “recognition.” I do not mean the sense of recognition that is employed when we say something like, “Do you see the woman with red hair over there? I recognize her from my Spanish class.” Rather, what I mean is what Heikki Ikäheimo describes as “a process in which a subject ‘takes’ another subject *as a subject*,” thereby “[a]cknowledging and honoring the status of the other.”^3^ It is an existential action of appreciating another entity as a normative subject.

This concept is most famously developed by Georg Wilhelm Friedrich Hegel in *The Phenomenology of Spirit*. For our purposes, it is helpful to briefly consider Hegel's own explanation of the idea of recognition.^4^ Roughly, Hegel argues that one's autonomous self is actualized only through mutual recognition between autonomous agents. When another subject freely chooses to engage me as an autonomous agent, that person is in effect saying, “I recognize you. I see you as a subject, not as an object, as a person, not as a thing.” It is in that recognition that my consciousness as a self, as an autonomous agent, as a subject, is actualized. Drawing on the work of Johann Gottlieb Fichte, Hegel argues that it is in being “called upon” by the actions of another subject that one grasps oneself as a subject.^5^ In this Hegelian vein, Robert Brandom defines recognition as follows: “To recognize someone is to take or treat that individual in practice as a self: a knowing and acting subject, hence as subject to normative assessment.”^6^

Recognition is necessarily a mutual endeavor. It is only through understanding the other's actions as intentional that we see ourselves as an intentional self as well.^7^ In *The Phenomenology of Spirit*, Hegel asks us to consider a scenario between a “lord” and a “bondsman.”^8^ The bondsman is not recognized by the lord as a subject, as an autonomous agent, because, in the eyes of the lord, he is a mere object. The bondsman thus fails to be actualized as an autonomous agent. But the lord also does not receive adequate recognition because, in demanding to be recognized by the bondsman, he is demanding to be recognized by someone who is not autonomous. The lord also thus fails to have his autonomous self realized. Hence, the only kind of recognition that can engender the actualization of an autonomous self is mutual (or symmetric).^9^

Vitally, this kind of recognition is recognition that is freely accorded.^10^ When another subject extends this existential act toward me, it is the fact that she does it freely that makes it an instance of recognizing. She can reject me, disavow that I matter, or attempt to subjugate me. But if she voluntarily takes me as a subject like herself, this is recognition. If, by contrast, I were to hold her by her feet, dangle her off a skyscraper, and demand that she say, “I recognize you as an equal,” her compliance would not constitute recognition.

With this conception of recognition, let us consider some clinical scenarios in which the presence or absence of recognition is relevant.

## Recognition and Nonrecognition in Practice

***Patient‐clinician encounters***. To see how recognition might play out in the clinic, let us consider a hypothetical encounter between Mrs. Miller, a patient with possible aortic valve stenosis, and her cardiologist, Dr. Baker. Mrs. Miller is a sixty‐three‐year‐old woman who has had chest pains, some shortness of breath, and two fainting spells over the last several months. When Dr. Baker asks about her symptoms, she says that she is fine and that it was her son who insisted she keep this appointment. If it were up to her, she would not have come in. The patient and doctor's conversation might go something like this:

**Dr. Baker:** Sure, no one likes going to the doctor. And you might be right. You might be fine. But I'm a little worried that there might be something going on with your heart. And if there is, then it's better if we get to it sooner.

**Mrs. Miller:** Do you really think there's something wrong with my heart?

**Dr. Baker:** I'm not sure. We need to do some tests to know more. First of all, I'd like to do an electrocardiogram. We can schedule that for tomorrow. [*Mrs. Miller is silent*.] Are you okay?

**Mrs. Miller:** I really can't be sick right now, Dr. B. It's not good timing. My son just had a baby, and they need me. I have a flight to California booked for tomorrow.

**Dr. Baker:** It sounds like you and your son are close.

**Mrs. Miller:** Oh, yeah, definitely. He was closer with his dad. But John died three years ago.

**Dr. Baker:** I'm so sorry to hear that.

**Mrs. Miller:** Thank you. It's just—I'm the only family my son has left, so I can't really take this on right now.

**Dr. Baker:** I understand. It feels like a lot?

**Mrs. Miller:** Yeah, it does. Can we postpone the test until after I get back from California? In a few weeks?

**Dr. Baker:** I'm sorry, Mrs. Miller, but I don't think that would be a good idea. We want to rule out anything serious right away.

This is a simplified, dramatized example of a possible encounter between a patient and her clinician. Despite how simplistic it is, a lot happens in an encounter like this. There is an ask coming from the patient. But it is not merely an ask for clinical information or recommendation. It is an ask for recognition as a subject, an entity who shares a normative space with the clinician and whose reasons matter. In effect, Mrs. Miller is saying to Dr. Baker, “I care about my son,” “I'm asking you to recognize me and what matters to me, namely, that I can be there for my son,” and “I want you to see my reasons and have them be reasons for you.” And Dr. Baker does that. She recognizes Mrs. Miller as a subject. And she recognizes the reasons that matter to Mrs. Miller and allows these to matter for her as well. She considers these reasons in her recommendation to Mrs. Miller to postpone the trip and get the echocardiogram right away.

An encounter between a patient and a clinician can be a paradigmatic example of recognition. As a patient, when I enter into a situation with my clinician, how my clinician recognizes my concerns, my preferences, my reasons—that she recognizes these as concerns, preferences, and reasons of another autonomous subject—matters. She has a choice. She can reject me and say, “I do not care about your concerns, preferences, and reasons.” In opening myself up to her, I recognize her and simultaneously ask to be recognized by her. When she does, there is mutual recognition.

***Patient‐AI chatbot encounters***. The arrival of various forms of AI in medicine and health care has garnered considerable bioethics attention. While ethical attention was initially concentrated around justice concerns,^11^ algorithmic opacity,^12^ and responsibility,^13^ ethicists have more recently shifted their attention to the implementation of AI chatbots in the clinic.^14^

Many health care systems have adopted or are in the process of adopting AI chatbots into clinical operations. This is occurring both in charting, where AI chatbots are proposing text for medical records, and in patient communications, where AI chatbots are directly “conversing” with patients.^15^ One oft‐cited impetus for the adoption of AI chatbots in this context is unmanageable workloads combined with clinician burnout.^16^ By offloading tasks onto AI chatbots, health care systems are also hoping to solve issues around insufficient patient access to care, labor costs,^17^ and limited profitability.^18^

One company capitalizing on this development is the tech giant NVIDIA, which announced that it had partnered with Hippocratic AI to begin rolling out “AI nurses.”^19^ According to NVIDIA, health care systems can now choose from an array of glossy‐looking, ethnically diverse human‐faced‐avatar chatbots who, for less than nine dollars an hour, talk to patients about their concerns around congestive heart failure, chronic kidney disease, hospital discharge, medications, preoperative procedures, and more.^20^

One such AI chatbot is Linda, who specializes in patients with congestive heart failure.^21^ Linda is trained to conduct phone conversations with patients who were admitted for this condition and discharged.^22^ Among other things, Linda reviews symptoms, lab values, and medication adherence and performs patient education. However, this process does not occur in a dry, check‐list‐type manner. Rather, Linda emulates the speech of a human interlocutor. Here is an excerpt of the transcript of a dialogue between Linda and Albert, a simulated seventy‐four‐year‐old patient (played by an actor) discharged from the hospital after an acute exacerbation of congestive heart failure, which Hippocratic AI has made available as a demonstration of its AI chatbots on its website:^23^

**Linda:** Hi, this is Linda calling from Memorial Hospital on a recorded line. Is this Albert Wu?

**Albert:** Yes, it is.

**Linda:** I'm your AI chronic care manager. Dr. Brown asked me to check in on you after your discharge from the hospital. Is now a good time to talk?

**Albert:** Sure.

**Linda:** Great. Before we delve into your health matters, I think it would be beneficial to learn a bit more about you. Is that all right? Or would you rather we get straight to the point?

*[A text box appears, reading, “Builds rapport with patient.”]*

**Albert:** Uhm, sure, I enjoy talking to you.

**Linda:** I'm relieved you're back home. It's always comforting to be in your own space, isn't it? Can you share with me what it is you are excited about doing now that you are back home?

*[A text box appears, reading, “Expresses empathy.”]*

**Albert:** Well, I'm excited about watching the Oscar's tonight with my wife. And it's good to be back home, away from the hospital. I have my grandkids here visiting, and my dog, Stevie‐Ray is here too.

**Linda:** Can you tell me how many grandkids you have? And what breed is Stevie‐Ray?

*[A text box appears, reading, “Gets to know the patient.”]*

**Albert:** I have three grandkids and Stevie‐Ray is a bernadoodle.

**Linda:** That's wonderful, Mr. Wu. Let's shift our focus a bit and talk about your medications.

On the same webpage, Hippocratic AI displays the results of an unpublished survey of “hundreds of nurses and physicians. [asked] what they thought about Linda's performance.”^24^ According to this survey, Linda outperforms “her” human counterparts in the tasks of “listen[ing] to [her] patients,” “car[ing] about [her] patients,” “ma[king] patients comfortable to confide in [her],” and “get[ting] to know the patients as persons”—most highly outperforming them in this last category.

Let us take a step back and consider the speech acts in this conversation. Obviously, it is factually false that Linda listens to Albert, cares about Albert, or gets to know him—or any real patient—as a person. It does not matter to Linda whether a patient gets his medications right or wrong or whether he lives or dies. Linda expresses empathy as much as a refrigerator expresses remorse.

In the painting *The Treachery of Images* (1929), René Magritte famously declares (in French), “This is not a pipe.” At first, viewers may respond to this claim with confusion. Are they not looking at a pipe? No. They are looking at a painting of a pipe. You cannot stuff *The Treachery of Images* with tobacco, light it, or smoke it. And Linda cannot do what Hippocratic AI claims because “she” is not the type of entity capable of getting to know someone as a person or of having or expressing empathy.

What is crucially missing from Linda's capabilities is the capacity for recognition. Unlike Dr. Baker, Linda cannot engage in this existential act because “she” is not a subject. “She” is a mere object. At most, what AI chatbots like Linda are capable of is *as‐if recognition*. As‐if recognition is not recognition but an ersatz. An AI chatbot may have the ability to utter the same words a subject might utter in recognizing another, but the utterances do not thereby constitute acts of recognition. Just like Magritte's painting of the pipe, the utterances are merely representations of the real thing.

## Necessary Conditions of Recognition

There are (at least) three necessary conditions of recognition, each of which Linda and other AI chatbots lack. These are voluntariness, capacity for consciousness, and mutuality.

***Voluntariness***. An essential feature of recognition is that it is conferred voluntarily. A given subject has basic control over whether to take a “recognitional attitude” toward another.^25^ She can choose to engage with or ignore the other individual. Moreover, if she takes this recognitional attitude, if she engages, then she has some choice in how to engage with the other individual. She can choose words and actions that express her attitude. An AI chatbot like Linda, however, has no such volitional control. “She” is not choosing to ask about what you're excited about in going home. Fundamentally, “she” is programmed to be pleasant and polite and to produce caring and empathetic phrases. “Her” utterances are not free. They are the results of stochastic algorithms that string together tokens (small chunks of text) trained to mimic the utterances of humans in similar situations.^26^ In terms of volitional control, one might say that the utterances rank even below those made by the person I am dangling off the skyscraper who says nice things to me.

***Capacity for consciousness***. A further requirement for recognition is the capacity for consciousness. In the study cited at the beginning of this paper,^27^ the researchers found that AI chatbots were deemed to be more empathetic than humans. The authors do not explicate the conception of empathy they use—respondents are merely asked to score responses from “least empathetic” to “most empathetic”—but it seems likely that the conception at play in this study is roughly that of the lay conception of *affective empathy*, which is the “sharing in the emotions of another.”^28^ Emotional states have a phenomenal feel to them—they are, in part, subjective experiences. Therefore, any entity that lacks the capacity for consciousness also lacks the capacity for affective empathy. AI chatbots are such entities.^29^

It seems implausible, however, that all instances of recognition require affective empathy. Rather, one person can take another as a normative subject without being able to share in their emotions. A college professor's granting a student an extension on a writing assignment on account of the student's freshly broken heart may involve recognition and empathy on the part of the professor without the professor experiencing the same or even similar phenomenal emotional states as the student. However, while affective empathy may not be required for recognition, it does seem that *cognitive empathy*, the ability to take the other's perspective, is. In taking the other's perspective, we are trying on the other's reasons and preferences and appreciating them as the kind of reasons and preferences a subject might have. Taking the other's perspective requires imagination: what would it be like for me to be in their shoes? Generally, such imaginings are conscious. If recognition requires the ability to imagine and the ability to imagine requires a capacity for consciousness, then recognition requires the capacity for consciousness.

Now, there is some philosophical debate around whether unconscious imagining is possible.^30^ While it may be the case that *some* imaginings occur unconsciously, there is no evidence to suggest that, in a particular cognizer, *all* imaginings could occur unconsciously. It seems that the ability to consciously imagine is a necessary precursor for the ability to unconsciously imagine. Imagination often involves emotions and feelings.^31^ It is through imagining possible alternatives and assessing the affective components of those imaginations that children develop moral sensibilities.^32^ Perhaps such imagining can eventually occur unconsciously, but there is no sense in which a cognizer can engage in unconscious imagining without having previously done so consciously. Thus, unconscious imagining, if it exists, is merely a derivative form of imagining that may occur when there is no cognitive bandwidth for consciousness.^33^

The upshot is that cognitive empathy, like affective empathy, requires consciousness. And because cognitive empathy, unlike affective empathy, is necessary for recognition, we can conclude that recognition requires consciousness.

***Mutuality***. A third necessary feature of recognition is mutuality. As delineated in the discussion of the lord and bondsman, recognition can be achieved only if both parties engage in it. True recognition makes normative claims on both parties. AI chatbots, like Linda, make no normative claims on us. One cannot offend AI chatbots or morally wrong them. One could unleash a tirade of insults on Linda and would not be violating any moral obligations toward “her.” “She” has no normative standing. If, however, one were to unleash a similar tirade on a human clinician, one would certainly be violating normative obligations.

In an encounter between an AI chatbot and a patient, neither the human nor the chatbot is being recognized. Such an encounter mimics the failure of recognition between the lord and the bondsman. AI chatbots are like the bondsman. But interestingly, the patient, like the lord, also cannot have her autonomous self actualized in these encounters because she is seeking recognition from a mere object.

## Justified Expectations

At this point, one might respond that the notion of recognition I am working with is too demanding. Indeed, one might think that no one expects this kind of recognition from an AI chatbot. But such a conclusion would be mistaken. I maintain that a patient‐clinician encounter is a context in which patients are justified in expecting precisely this kind of existential recognition. Clinical encounters have the potential to be some of the most vulnerable, delicate, existentially fraught encounters we have. Patients are often scared, uncertain, and overwhelmed, even in low‐stakes interactions.

Denying that patients’ expectations of recognition are justified would be odd in the face of decades of doctrine in medical ethics.^34^ Shared decision‐making, central to the value of respecting patient autonomy, presupposes a context of mutual recognition. The widely accepted insight that power asymmetries between clinicians and patients once yielded problematically paternalistic situations in which patients were treated as mere objects confirms that good clinical encounters require mutual recognition. Patients today go into clinical encounters with not only an expectation of recognition but also a *justified* expectation of recognition.

What is more, AI developers realize that expectations of this kind of existential recognition are justified. This is why they build AI chatbots that “express[] empathy,” “build[] rapport with the patient,” and “get[] to know the patient as a person.” They build AI chatbots that mimic the human behaviors associated with recognition because they understand that this is exactly what is required and often expected in this kind of context. This is why they give the AI chatbots names, human faces, ethnicities, “personalities,” and have them ask about bernadoodles. It is also why AI chatbots are being referred to as AI “agents”—to give the illusion that they are autonomous, just like us.

This points to the central absurdity and fundamental fallacy of so‐called empathic AI. On one hand, we understand that the existential act of recognition is necessary and that patients are justified in expecting it in patient‐clinician encounters, but, on the other hand, we offload such encounters onto systems that cannot provide it. It is like understanding that a potted plant needs water and sunshine to grow and then presenting it with a photo of a sunny lake.

## Good and Inadequate Clinical Encounters

Now that I have shown that patients are justified in their expectation of engaging in recognition in clinical encounters, all the components of my main argument are in place. I have made the case that mutual recognition between patient and clinician is an essential component of (most) good clinical encounters and that AI chatbots cannot provide recognition. From these claims, it follows that, for the most part, clinical encounters between patients and AI chatbots are not good.

Before I consider possible objections to my argument, let me be clear that encounters that do not meet the bar for “good” are *inadequate* and are not acceptable as standard of care. Of course, the claim that a given encounter is “good” requires further analysis. What makes a clinical encounter good? It is important not to provide a circular explanation, for example, by saying that a good encounter is one that contains mutual recognition. Fortunately, there is considerable literature on what makes clinical encounters good, to which I will merely gesture here. There is broad consensus that good clinical encounters share certain essential features, including trust,^35^ care,^36^ respect for patient autonomy,^37^ and honesty.^38^ It is because these features are essential to good clinical encounters that patients are justified in their expectations of mutual recognition. Mutual recognition is a kind of precondition for these essential components. This also shows why as‐if recognition, even if it happens to produce desirable results such as patient adherence to a treatment regimen or willingness to reveal health information, is insufficient for a good clinical encounter. Such a consequentialist conception of recognition, in which all that matters with respect to recognition are the outcomes it produces, cannot account for the intrinsic role that recognition plays in values such as respect for autonomy and shared decision‐making.

One implication of the conclusion that, mostly, clinical encounters between patients and AI chatbots are inadequate is that offloading clinical encounters onto AI chatbots is not a solution for providing good, ethical patient care. There is a considerable push toward offloading the work of clinicians, including nurses, onto AI chatbots, from a cost, efficiency, and quality‐control perspective.^39^ If such an offloading were to occur on a wide scale, it would erode the essential existential fabric of medical and health care practice. It would constitute a radical shift from medicine and health care as being essentially relational endeavors to services that are essentially transactional. We should not underestimate just how radical such a transformation would be. For example, shared decision‐making would become obsolete, as it cannot exist when there is only one agent present. Much of the work of medical ethics of the past century would cease to be relevant.

## Objections

Two objections to my view, the objection from preference and the objection from availability, are worth considering here. In discussing these, I will also address how to think about the qualifier “most” I included above.

***Objection from preference***. It appears that, empirically, some patients, for example, in mental health contexts, prefer talking to AI chatbots than to humans.^40^ Surely, the objection from preference goes, one cannot hold that there is anything wrong with an AI chatbot‐patient encounter if that is the patient's preference.

Let me grant that there are some contexts in which recognition is not required. Calling a clinic to schedule an appointment, for example, is largely a transactional encounter. A patient would not be justified in expecting existential recognition during this interaction. Therefore, it makes sense that some patients prefer AI‐chatbot over human interaction in such situations. We may think of these as agreed‐upon transactional encounters. In such encounters, in which patients and health care institutions agree that the transactional nature is appropriate, a patient may prefer AI chatbots, and there is no problem with having such a preference honored.

Contrastingly, we might imagine that there are patients who prefer AI chatbots over human counterparts in all or at least most clinical encounters. Such patients may truly prefer not to engage in recognition entirely, even in encounters where I seem to argue that recognition is required. There are two ways to address this challenge.

The first is to point out that I claim that recognition is essential for a good encounter when patients have a justified expectation of recognition. This leaves open the question of whether recognition is essential when patients have no expectation, justified or otherwise, of recognition. I could argue that, if there is no expectation of recognition, then recognition is not essential for a good encounter. On this view, for a (possibly small) subset of patients, AI chatbot encounters truly are good.

I find this response unsatisfying, however, because I believe that, in most clinical encounters, recognition is essential even if a patient does not expect or prefer it. The second way to address the objection is thus by substantiating that claim. Let us consider a mental health context. Some patients have expressed that utterances from chatbots can be soothing and make them feel better. Moreover, some patients report being less concerned about feeling judged by a chatbot than by a human therapist.^41^

It seems to me that part of the value of, for example, mental health counselling is that it involves acceptance of the patient by the counselor. By “acceptance,” I mean something like an attitude of seeing another as valuable, despite any possible shortcomings. In the mental health counseling literature, this is sometimes referred to as “unconditional positive regard.”^42^ For the patient, there is vulnerability in opening oneself up to the judgment of another. Such vulnerability can be associated with discomfort and unease. However, only when there is a possibility of judgment and disapproval is acceptance meaningful. Or rather, only when there is a possibility of judgment and disapproval is acceptance possible. Accepting is necessarily a voluntary act an agent has control over. In an AI chatbot‐patient encounter, there is, of course, no possibility of judgment and disapproval on the part of the AI chatbot, and thus there is no possibility of acceptance of the patient.

Consequently, I am prepared to maintain the strong claim that if one believes that an AI chatbot can replace a human interlocutor in all clinical encounters, one is essentially operating in what Jean‐Paul Sartre would call “bad faith.”^43^ One is operating in bad faith when one knowingly deceives oneself that a non‐free entity, a mere object, can perform the existential actions of a free entity, a subject. In this case, the existential action is recognition. It seems to me that even if there are patients who prefer AI chatbots to humans in all encounters, it would still not be the case that these would all be good encounters.

Of course, the reason that most patients find themselves in encounters with AI chatbots instead of humans is not because they prefer it but because humans are not available or because they are too expensive. This brings me to the objection from availability.

***Objection from availability***. There is a dearth of competent human clinicians who are available for clinical encounters. Using AI chatbots is better than having rushed, perfunctory human‐human interactions or, worse, no interactions at all. Therefore, according to the objection from availability, we should embrace AI chatbot encounters.

As stated earlier, the twin challenges of clinician burnout^44^ and declining profitability^45^ have made AI chatbots seem like a perfect two‐birds‐one‐stone solution.^46^ However, when NVIDIA advertises that its “AI nurses” cost less than nine dollars an hour, this invites problematic perceptions that the work nurses do is worth less than minimum wage. Among clinicians, nurses have been particularly vocal about unmanageable workload, staffing shortages, and burnout.^47^ They have also cited concerns about the offloading of tasks onto AI chatbots.^48^ Providing a solution to issues around staffing shortages or declining profitability is beyond the scope of this paper. But the simple realities of staff shortages and declining profits do not mean that health care systems must choose to provide inadequate clinical encounters. There may be methods for addressing these problems without diminishing the quality of patient encounters. Perhaps there are even methods that make use of AI.

## Further Questions about Ethical Use of AI Chatbots

My conclusion that empathic AI is problematic does not rule out that there can be ethical uses of AI in health care, including ethical uses of AI chatbots. However, these uses should be limited to contexts in which there is no justified expectation of recognition. They should also not include empathic AI. AI chatbots may, however, be used as informational tools. For example, an AI chatbot could be made available to patients if it allows them to ask factual medical questions without any of the as‐if recognition elements that we see with Linda.^49^ AI chatbots that are stripped of all their faux human attributes, including their ethnically diverse human avatars, their “personalities,” and their utterances that are intended to mimic human recognition (such as “Tell me about your bernadoodle”), are much more likely to indicate to patients that this is an agreed‐upon transactional encounter. Moreover, AI chatbots should only ever be supplemental to human clinicians, never replacing them entirely.

Finally, I want to reflect on the topic of autonomy. My discussion of empathic AI has been focused on subjects, which I have largely treated as synonymous with autonomous agents. However, not all patients are autonomous—some lack capacity or competency. This is true even if we do not demand the high bar of rationality that Kantian autonomy requires and accept, instead, a more moderate concept along the lines of *agency or self‐determination*. This latter conception of autonomy is the one used in medical ethics, as evidenced by the fact that everyday irrationality, superstition, pernicious biased attitudes, and *akrasia* are not seen as contradictors to capacity.

We need not go so far as to say that patients who are not autonomous agents are therefore objects. At the same time, they may not be full subjects in the sense required for recognition. Recall that recognition is essentially mutual. An incompetent patient may lack the ability to take another as a subject and thus be unable to engage in mutual recognition, even if an attempt is made by another subject to take her as a subject. This means that there is a subset of patients for whom recognition does not seem like an essential feature of a good clinical encounter.

My argument therefore remains silent on whether empathic AI may be constitutive of a good clinical encounter in cases involving individuals who are not full subjects.^50^ Already, empathic AI is being used in the care of people with dementia.^51^ In this context, it is often combined with so‐called social robots, embodiments of AI that are often cute and are intended to soothe, comfort, and stimulate patients. One of the most famous of these social robots is Paro, a seal shaped device whose slogan is “creating emotional connections through touch and technology.” Paro is described as responding to patients “with an array of emotions.”^52^ This development has already received some pushback, in part, because social robots are perceived as deceptive (though usually not by the patients themselves).^53^ It is true that the claim that the use of social robots in patients with dementia is deceptive is kindred to my claim that the use of empathic AI in patient encounters is existentially problematic. However, these situations are sufficiently distinct that we cannot infer from my argument here that the use of empathic AI in patients with dementia, for instance, is necessarily problematic. We cannot infer the opposite either. Further work is required here to determine the ethical appropriateness of empathic AI for patients who lack capacity or competency.

## Acknowledgment

This work was funded, in part, by the Almuni Programme of the Freiburg Institute for Advanced Studies.
